# In Vitro and In Silico Studies of Maculosin as a Melanogenesis and Tyrosinase Inhibitor

**DOI:** 10.3390/molecules30040860

**Published:** 2025-02-13

**Authors:** Yang Xu, Xuhui Liang, Hyeon-Mi Kim, Chang-Gu Hyun

**Affiliations:** 1 Department of Chemistry and Cosmetics, Jeju Inside Agency and Cosmetic Science Center, Jeju National University, Jeju 63243, Republic of Korea; iamxuyang1990@gmail.com (Y.X.); lxh03036@naver.com (X.L.); gusal1388@naver.com (H.-M.K.); 2 Department of Beauty and Cosmetology, Jeju National University, Jeju 63243, Republic of Korea

**Keywords:** B16F10, maculosin, melanogenesis, tyrosinase, molecular docking, molecular dynamics simulations

## Abstract

The investigation of melanogenesis and tyrosinase inhibitors is essential for developing safe and effective natural compounds to treat pigmentation disorders. This study aimed to evaluate the effects of maculosin, a cyclic dipeptide composed of tyrosine and proline, on melanin production and tyrosinase activity using the B16F10 melanoma cell model, while elucidating its mechanism of action through molecular docking and molecular dynamics (MD) simulations. Experimental results demonstrated that maculosin inhibited intracellular melanin content and tyrosinase activity in a concentration-dependent manner in B16F10 melanoma cells. Molecular docking analyses revealed that maculosin exhibited high binding affinities with mushroom tyrosinase (mTYR), tyrosinase-related protein 1 (TYRP1), and *Bacillus megaterium* tyrosinase (*Bm*TYR) with binding energies of −7.7, −6.8, and −7.5 kcal/mol, respectively. Furthermore, MD simulations confirmed the structural stability and dynamic flexibility of maculosin–protein complexes, as indicated by RMSD, RMSF, Rg, SASA, hydrogen bond interactions, PCA, and DCCM analyses. Binding free energy calculations using the MM/PBSA method showed that maculosin exhibited binding energies of −28.76 kcal/mol with mTYR and −22.23 kcal/mol with TYRP1, outperforming standard co-crystal inhibitors such as tropolone (−12.47 kcal/mol) and kojic acid (−12.73 kcal/mol). Critical residues, including VAL-283 and HIS-263 in mTYR and HIS-381, GLY-389, and THR-391 in TYRP1, were identified as key contributors to maculosin binding, corroborating molecular docking findings and displaying strong correlations in DCCM analyses. Collectively, these results suggest that maculosin is a highly promising candidate for the treatment of pigmentation disorders, offering significant inhibitory effects on melanogenesis and tyrosinase activity.

## 1. Introduction

Melanin, a natural pigment found in the skin, hair, and eyes, plays a critical role in protecting the body from ultraviolet (UV) radiation by absorbing UV light and neutralizing free radicals, thereby reducing UV-induced skin damage and lowering the risk of skin cancer [1]. Melanin biosynthesis, or melanogenesis, is a multi-step enzymatic process initiated by the enzyme tyrosinase (TYR), which catalyzes the conversion of tyrosine into DOPA, ultimately leading to the formation of melanin precursors and melanin itself [2]. This pathway involves several enzymes, including tyrosinase-related proteins (TRP-1 and TRP-2), which interact to regulate melanin production [3]. Additionally, transcription factors such as MITF (microphthalmia-associated transcription factor) play a pivotal role in modulating the expression of genes involved in melanogenesis [4]. Intercellular signaling between melanocytes, keratinocytes, and fibroblasts further influences melanin production [5,6].

The dysregulation of melanin synthesis can result in pigmentation disorders, including hyperpigmentation, melasma, freckles, and age spots, which are common dermatological concerns [7,8]. As tyrosinase is a key enzyme in this process, its inhibition has become a widely adopted strategy for developing skin-lightening agents [9,10]. Among natural compounds, diketopiperazines (DKPs), the smallest cyclic peptides, have attracted significant interest due to their diverse bioactivities and structural simplicity. DKPs, formed by the condensation of two α-amino acids such as Tyr, Pro, Ala, His, and Gly, are synthesized by various microorganisms and are classified based on their biosynthetic enzymes: nonribosomal peptide synthetases (NRPSs) [11,12], cyclodipeptide synthases (CDPSs) [13,14], and arginine-containing CDPSs (RCDPSs) [15]. Further structural modifications of DKPs are achieved by tailoring enzymes such as oxidoreductases, methyltransferases, and prenyltransferases, enhancing their biological activities [16,17,18].

Several DKPs, including albonoursin [19], mycocyclosin [20], pulcherriminic acid [21], bicyclomycin [22], and nocazine derivatives [23], exhibit a wide range of bioactivities, including antibacterial [24], antifungal [25], antiviral [26], antitumor [27], and anti-inflammatory effects [28]. Despite extensive research on the biological activities of DKPs, their potential in inhibiting melanin synthesis and tyrosinase activity remains underexplored. Maculosin, a DKP first isolated from the Caribbean sponge *Calyx* cf. *podatypa* [29], is also commonly found in microbial secondary metabolites from species such as *Bacillus*, *Pantoea*, and *Paenibacillus* [30,31]. Known for its antioxidant [32], anti-inflammatory [33], and antifungal properties [34], maculosin has been reported to inhibit mushroom tyrosinase activity [35], highlighting its potential as a candidate for skin-whitening applications.

**Figure 1:**
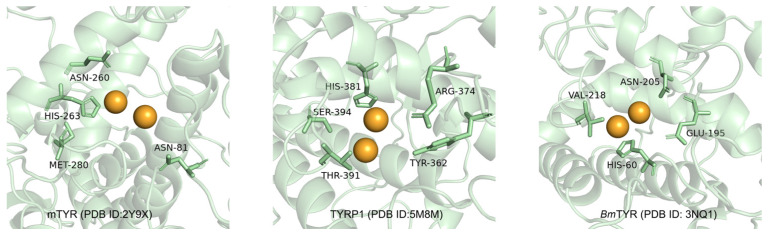
Key amino acids in the active cavity of mTYR, TYRP1, and *Bm*TYR from molecular docking studies.

The B16F10 mouse melanoma cell line, characterized by high melanin production and stable tyrosinase activity, serves as a reliable in vitro model for studying melanogenesis due to its similarity to human epidermal melanocytes [36]. Previous molecular docking studies have elucidated key binding interactions between tyrosinase and its inhibitors, providing insights into the design of effective therapeutic agents. Notably, tyrosinase from various sources, including mushroom tyrosinase (mTYR), tyrosinase-related protein 1 (TYRP1), and *Bacillus megaterium* tyrosinase (*Bm*TYR), has been extensively studied (Figure 1). Critical residues such as HIS-263 in mTYR [37,38] and HIS-381 in TYRP1 [39,40] have been identified as essential for ligand binding, offering a structural basis for designing tyrosinase inhibitors.

This study aimed to evaluate the inhibitory effects of maculosin (Figure 1) on melanogenesis and tyrosinase activity in B16F10 cells and to elucidate its binding mechanisms through molecular docking and molecular dynamics (MD) simulations. These findings provide a foundation for developing maculosin as a natural, low-toxicity candidate for skin-whitening agents and treatments for pigmentation disorders.

## 2. Results and Discussion

### 2.1. Biological Evaluations

#### 2.1.1. Cell Viability

**Figure 2:**
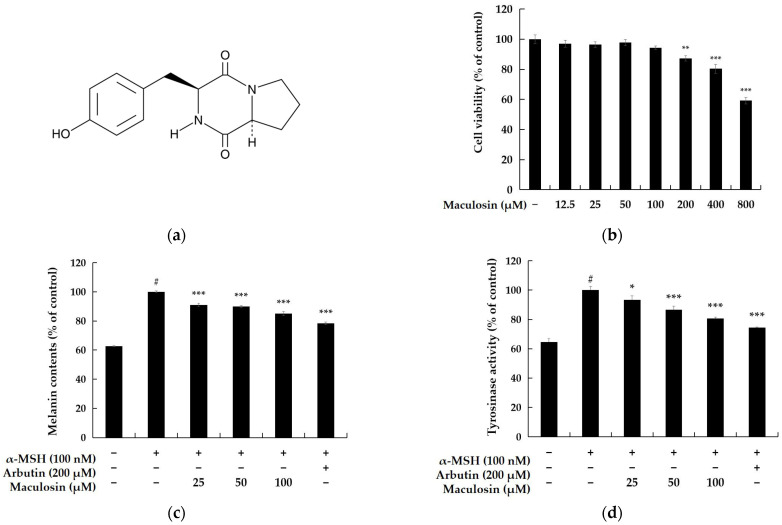
Effects of maculosin on cell viability, melanin content, and tyrosinase activity in B16F10 melanoma cells. (**a**) Chemical structure of maculosin. (**b**) Cell viability determined by the MTT assay expressed as a percentage relative to the untreated control group. (**c**,**d**) Effects of maculosin on melanin content and tyrosinase activity in α-MSH (100 nM)-stimulated cells with arbutin used as a positive control. Data are presented as mean ± SD from three independent experiments. Statistical significance: # *p* < 0.001 vs. the unstimulated control group. * *p* < 0.05, ** *p* < 0.01, *** *p* < 0.001 compared to the respective controls.

Cell viability was evaluated in B16F10 melanoma cells to identify a compound concentration that does not adversely affect cell viability. The MTT assay, a colorimetric method measuring metabolic activity, was used for this assessment. The results indicated 82.27% viability at 200 µM and 94.27% at 100 µM relative to the untreated control group. Based on these findings, subsequent experiments were performed using concentrations below 100 µM to minimize cytotoxicity while investigating the compound’s biological activity (Figure 2b).

#### 2.1.2. Melanin Contents and Tyrosinase Inhibiton Activity of Maculosin

Melanin synthesis and tyrosinase activity were assessed in B16F10 melanoma cells to examine the effects of α-MSH and maculosin on melanogenesis. Cells were treated with α-MSH (100 nM) and varying concentrations of maculosin, including 100 µM, for 72 h. Melanin content and tyrosinase activity were subsequently measured to evaluate the impact of maculosin on B16F10 cells. The results demonstrated that treatment with 100 µM maculosin reduced melanin production by 14.84% and inhibited tyrosinase activity by 19.35% compared to the untreated control group. These findings suggest that maculosin may suppress melanogenesis by inhibiting tyrosinase activity, thereby reducing melanin synthesis.

### 2.2. Molecular Properties and Drug Likeness

The comparative analysis of maculosin, kojic acid, tropolone, and arbutin reveals certain ADMET advantages of maculosin (Table S1). Tropolone showed the highest Caco-2 permeability (1.558 cm/s) and intestinal absorption (98.108%), which wa followed by kojic acid (0.637 cm/s, 93.152%). Maculosin, with lower permeability (−0.009 cm/s), had a higher absorption rate (66.287%) than arbutin (38.027%), indicating potential for oral bioavailability. Maculosin’s plasma protein binding (PPB) (44.6%) was comparable to that of tropolone (47.9%) and superior to kojic acid (23.3%), while it was slightly lower than that of arbutin (54.2%), showing effects similar to tropolone and arbutin in terms of PPB. Its volume of distribution (0.204 L/kg) was significantly higher than that of the other compounds, reflecting superior tissue distribution. Unlike tropolone, maculosin does not penetrate the BBB, potentially avoiding neurotoxicity risks. Furthermore, maculosin exhibited a slower clearance rate (0.264 mL/min/kg) than kojic acid and arbutin, aiding steady-state maintenance, while its shorter half-life (1.398 h) reduces the risk of drug accumulation. Maculosin showed no Ames toxicity, hERG inhibition, or skin sensitization, but its hepatotoxicity requires further evaluation. Importantly, its LD_50_ (1.674 mol/kg) was within a comparable range to other compounds, lower than tropolone and kojic acid, and slightly higher than arbutin. Overall, maculosin showed favorable characteristics in tissue distribution, plasma protein binding, and steady-state concentration maintenance with advantages over kojic acid in binding and distribution, tropolone in potential neurotoxicity, and arbutin in absorption. Furthermore, maculosin did not exhibit hERG toxicity or significant metabolic enzyme inhibition, suggesting a favorable safety assessment.

Maculosin exhibited superior drug-likeness compared to the other three compounds, satisfying all major criteria, including RO5, the Ghose filter, Veber rule, and Egan rule (Table S2). In contrast, kojic acid, tropolone, and arbutin did not fully meet the Ghose filter requirements. Specifically, both kojic acid and tropolone had three violations concerning molecular weight, refractivity, and atom count, while arbutin had one violation related to WLOGP. Although the hepatotoxicity of maculosin required further investigation, its overall pharmacokinetic properties suggested significant potential for application, making it a promising candidate compared to the other three compounds.

### 2.3. Molecular Docking Simulation

We have previously reported the methodological validation for the interactions of mushroom tyrosinase (mTYR, PDB ID: 2Y9X) and tyrosinase-related protein 1 (TYRP1, PDB ID: 5M8M) [41]. For *Bm*TYR (PDB ID: 3NQ1), re-docking of the co-crystal inhibitor, kojic acid, showed minimal conformational changes (RMSD = 0.317 Å), confirming the validity of the methodology (Figure S1). The binding energy between the co-crystal ligand and *Bm*TYR was calculated as −5.9 kcal/mol, suggesting a strong ligand–protein interaction.

The docking studies revealed that maculosin demonstrated strong binding affinity for all three tyrosinase types (mTYR, TYRP1, and *Bm*TYR) with binding energies of −7.7, −6.8, and −7.5 kcal/mol, respectively. Importantly, maculosin interacted with these enzymes through a combination of hydrogen bonding, π–π stacking, π–sigma, π–alkyl, and carbon–hydrogen interactions, suggesting its potential as a comparatively effective inhibitor.

**Figure 3:**
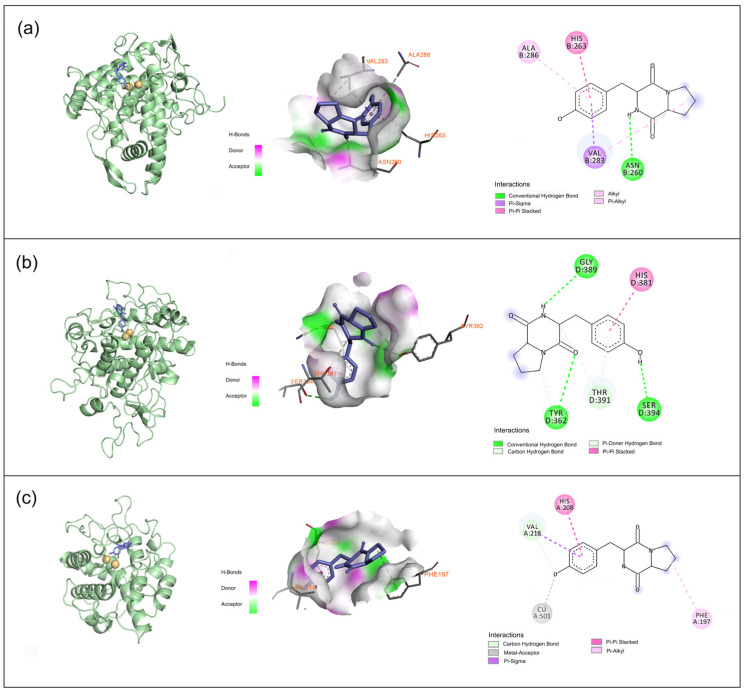
Binding interactions of tyrosinase protein with maculosin: (**a**) mTYR−maculosin; (**b**) TYRP1−maculosin; (**c**) *Bm*TYR−maculosin.

The docking binding energy of the mTYR–maculosin complex was −7.7 kcal/mol (Figure 3a). Maculosin’s NH group formed a hydrogen bond with ASN-260, while its phenyl ring engaged in various π interactions with surrounding residues (HIS-263, VAL-283, and ALA-286). The pyrrolidine ring also contributed to the binding via alkyl interactions with VAL-283. In the TYRP1–maculosin complex, with a docking binding energy of −6.8 kcal/mol (Figure 3b), maculosin formed hydrogen bonds with GLY-389, TYR-362, and SER-394 through its NH group, along with its adjacent carbonyl and hydroxyl groups, respectively. The phenyl ring of maculosin also formed π–π stacking interactions with HIS-381, suggesting that maculosin may have influenced the enzyme’s activity by stabilizing these interactions. In the *Bm*TYR–maculosin complex, the docking binding energy was −7.5 kcal/mol (Figure 3c). Maculosin exhibited similar binding modes, including carbon–hydrogen and π–sigma interactions with VAL-218 and π–π stacking interactions with HIS-208. Additionally, the pyrrolidine group formed π–alkyl interactions with PHE-197, the hydroxyl group acts as a metal acceptor, interacting with the copper ion, further stabilizing the ligand–enzyme complex. Overall, maculosin demonstrated strong binding to all three tyrosinase types, suggesting its potential as a candidate for modulating tyrosinase-related activities.

### 2.4. Molecular Dynamics (MD) Simulation

#### 2.4.1. Structural Stability of the Complexes

**Figure 4:**
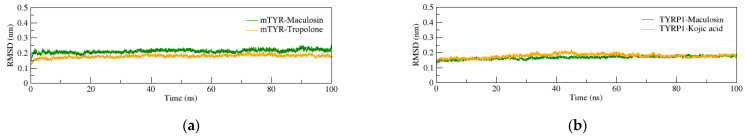
RMSD analysis of MD simulations for protein-ligand complexes. (**a**) mTYR–ligand; (**b**) TYRP1–ligand.

Considering maculosin’s demonstrated inhibitory activity against tyrosinase, semi-flexible molecular docking was conducted to evaluate the binding affinity of ligand–protein complexes. To further investigate the dynamic stability and binding behavior of the ligand–protein systems, 100 ns MD simulations were performed on both mTYR and TYRP1 complexes. For the mTYR–ligand complex (Figure 4a), the co-crystal ligand was tropolone. The root-mean-square deviation (RMSD) of the mTYR–tropolone complex fluctuated between 0.15 and 0.20 nm, while the RMSD of the mTYR–maculosin complex was slightly higher, ranging from 0.15 to 0.25 nm. Despite this minor increase, the RMSD values of the mTYR–maculosin complex consistently remained within the acceptable range of 0.1–0.3 nm, with a stable trajectory throughout the simulation, indicating robust complex stability. For the TYRP1–ligand complex (Figure 4b), the co-crystal ligand was kojic acid. The RMSD profiles of the TYRP1–kojic acid and TYRP1–maculosin complexes were nearly identical, maintaining low and stable RMSD values throughout the simulation. This similarity suggests a comparable level of stability for both complexes. These findings underscore maculosin’s potential as a stable tyrosinase inhibitor in both mTYR and TYRP1 systems.

**Figure 5:**
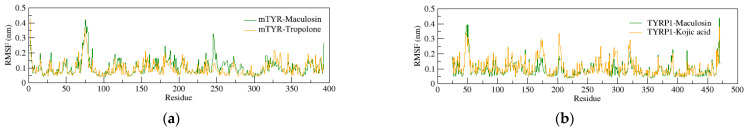
RMSF analysis of MD simulations for protein–ligand complexes. (**a**) mTYR–ligand; (**b**) TYRP1–ligand.

In the mTYR–ligand complexes (Figure 5a), the root mean square fluctuation (RMSF) curves for the mTYR–tropolone and mTYR–maculosin complexes show considerable overlap with both complexes exhibiting minimal fluctuations in amino acid residues between positions 70 and 80. The RMSF values for both complexes remain below 0.45 nm, indicating that the binding of the compounds does not induce significant fluctuations in the amino acid residues of mTYR. This suggests a high degree of stability for both complexes.

In the TYRP1–ligand complexes (Figure 5b), the RMSF curves for the TYRP1–kojic acid and TYRP1–maculosin complexes show fluctuations below 0.4 nm in the amino acid residues spanning positions 45 to 55. At the protein’s edge (residues 455–466), the fluctuations approach 0.45 nm. Overall, the maculosin-containing protein complexes demonstrate stability comparable to that of the co-crystal inhibitors, suggesting similar binding and conformational stability.

**Figure 6:**
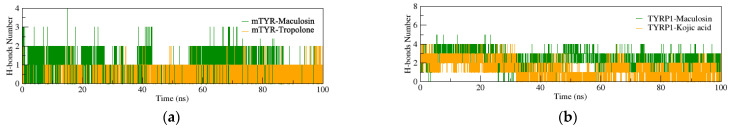
H-bonds analysis of MD simulations for protein–ligand complexes. (**a**) mTYR–ligand; (**b**) TYRP1–ligand.

To evaluate hydrogen bonding interactions at the binding sites, the number of key hydrogen bonds stabilizing the ligand–protein complexes was analyzed. In the mTYR–ligand complex (Figure 6a), maculosin formed stable hydrogen bonds (1–2 bonds) with the mTYR protein, indicating strong binding stability. In comparison, the co-crystal ligand tropolone maintained a consistent single hydrogen bond throughout the simulation. In the TYRP1–ligand complex (Figure 6b), maculosin formed three hydrogen bonds with TYRP1, while kojic acid stabilized with one to two hydrogen bonds. Overall, maculosin exhibited a higher number of hydrogen bonds than the co-crystal inhibitors, suggesting stronger binding interactions with both protein targets.

#### 2.4.2. Dynamic Structural Conformational Analysis of the Complexes

**Figure 7:**
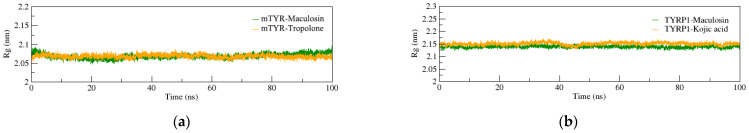
Rg analysis of MD simulations for protein-ligand complexes. (**a**) mTYR–ligand; (**b**) TYRP1–ligand.

The radius of gyration (Rg) measures the compactness of a complex structure with larger Rg values indicating expansion and smaller values suggesting a more compact system. In the mTYR–ligand complexes (Figure 7a), the Rg curves for both compound complexes overlap closely with fluctuations maintained within the range of 2.05–2.1 nm. This indicates a stable pattern with minimal deviation throughout the simulation. Similarly, in the TYRP1–ligand complexes (Figure 7b), Rg fluctuations for both compound complexes remain within the range of 2.15 ± 0.05 nm. These findings suggest that both mTYR and TYRP1 proteins form compact and stable complexes with both the co-crystal ligand and maculosin.

**Figure 8:**
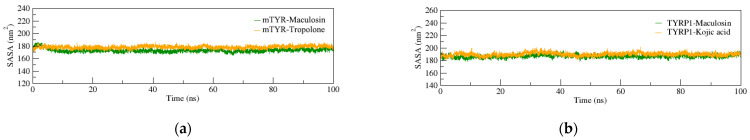
SASA analysis of MD simulations for protein–ligand complexes. (**a**) mTYR–ligand; (**b**) TYRP1–ligand.

The solvent-accessible surface area (SASA) is a key factor in evaluating protein folding and stability. Proteins with stable structures typically exhibit consistent SASA curves. In the mTYR–ligand complex (Figure 8a), the SASA curves for both compound complexes closely overlap, showing stable fluctuations throughout the simulation without significant deviations. Similarly, in the TYRP1–ligand complex (Figure 8b), the SASA curves exhibit good stability. Overall, the complexes formed by mTYR and TYRP1 with the co-crystal ligands and maculosin demonstrate stable structural characteristics.

**Figure 9:**
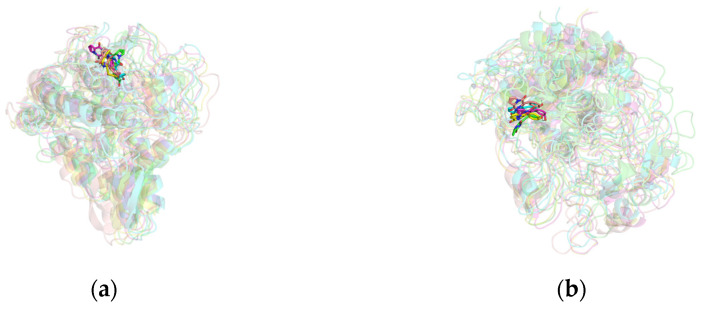
The conformational structures of the complex in MD simulations: (**a**) mTYR–maculosin; (**b**) TYRP1–maculosin. The configurations at different time points are represented as follows: 0 ns (green), 25 ns (blue), 50 ns (purple), 75 ns (yellow), and 100 ns (pink).

**Figure 10:**
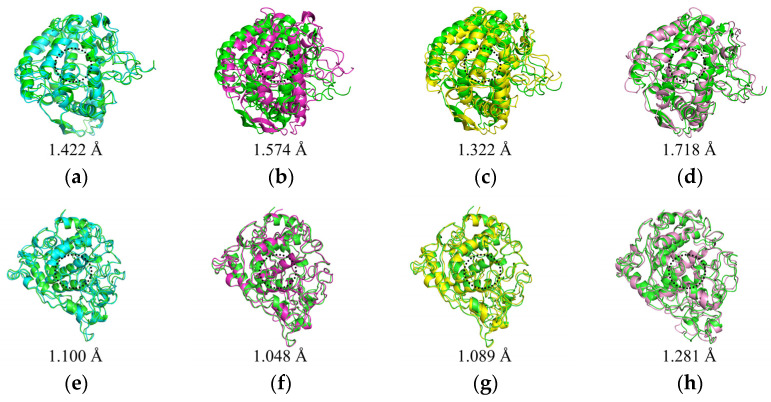
Comparison of initial and time-evolved structures in MD simulations. mTYR–maculosin: (**a**) 0–25 ns; (**b**) 0–50 ns; (**c**) 0–75 ns; (**d**) 0–100 ns. TYRP1–maculosin: (**e**) 0–25 ns; (**f**) 0–50 ns; (**g**) 0–75 ns; (**h**) 0–100 ns.

By analyzing the conformations of the complexes at five time points (0, 25, 50, 75, and 100 ns) during molecular dynamics simulations, the binding stability of the complexes was assessed. In the mTYR–ligand complex, both the co-crystal ligand tropolone [29] and maculosin (Figure 9a) consistently remained at the active binding site of the mTYR protein across all time points without significant changes, indicating high binding stability. Similarly, in the TYRP1–ligand complex, the co-crystal ligand kojic acid (Figure S2) and maculosin (Figure 9b) maintained proximity to the active site of the TYRP1 protein throughout the simulation. Overall, maculosin demonstrated favorable stability in complexes with both protein systems. Structural changes and stability during MD simulations were further evaluated by comparing the overlap of initial and time-evolved protein conformations using RMSD deviations. Upon ligand binding, mTYR exhibited RMSD deviations of 1.422 Å, 1.574 Å, 1.322 Å, and 1.718 Å at 25 ns, 50 ns, 75 ns, and 100 ns, respectively, as shown in Figure 10a–d. Similarly, TYRP1 showed RMSD deviations of 1.1 Å, 1.048 Å, 1.089 Å, and 1.281 Å at the same time points with maculosin, as depicted in Figure 10e–h. Importantly, beyond the significant conformational changes observed in the loop regions of mTYR and TYRP1 within the catalytic pocket, other secondary structural elements, including *α*-helices and *β*-sheets, also underwent notable rearrangements.

#### 2.4.3. Principal Component and Dynamical Residues Cross-Correlation Binding Analysis

**Figure 11:**
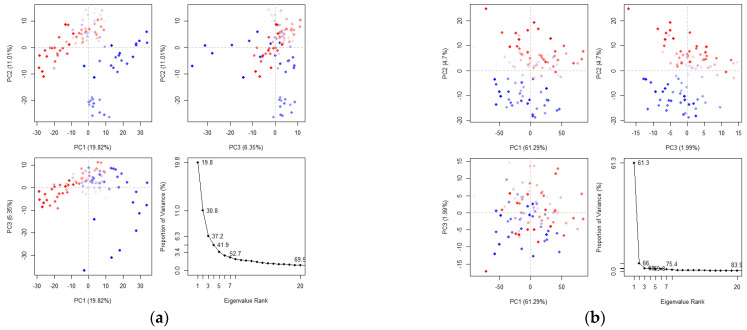
Principal component analysis of clusters by protein complex dynamics. (**a**) mTYR–maculosin; (**b**) TYRP1–maculosin.

Principal component analysis (PCA) reduces the complexity of high-dimensional molecular dynamics data by calculating the covariance matrix and identifying key motions that account for most of the variance. These motions are crucial for understanding protein function, stability, and behavior [42,43]. The axes represent different dimensions of the protein’s conformational space with each dot corresponding to a unique protein configuration from the simulation. The color gradient, ranging from blue to red, indicates the simulation’s progression: blue represents the initial timestep, white denotes intermediate states, and red corresponds to the final timestep. In the PCA analysis of the maculosin–mTYR protein complex (Figure 11a), the first three principal components (PC1, PC2, and PC3) explain 37.18% of the total variance. PC1 (19.82%) reflects large-scale conformational changes, capturing major structural rearrangements within the complex. PC2 (11.01%) represents intermediate-level motions, likely associated with local ligand–protein interactions, while PC3 (6.35%) accounts for smaller-scale fluctuations in specific protein regions. For the maculosin–TYRP1 protein complex (Figure 11b), the first three principal components account for 68.98% of the total variance with PC1 explaining 61.29%, PC2 4.7%, and PC3 1.99%. These results highlight the dominant structural dynamics captured by PC1 in the maculosin–TYRP1 complex compared to the maculosin–mTYR complex.

The PCA comparison revealed that the maculosin–TYRP1 complex exhibited more pronounced large-scale conformational changes than the maculosin–mTYR complex, as indicated by the higher contribution of PC1. This suggests differences in their interaction mechanisms. The TYRP1 complex displayed greater conformational flexibility and mobility, potentially enabling a more adaptive and tunable binding mode to optimize ligand–protein interactions.

The conformational changes induced by maculosin in mTYR and TYRP1 proteins were further analyzed using dynamic cross-correlation matrix (DCCM) analysis, which revealed both positive and negative amino acid correlation effects. The DCCM displayed correlations ranging from −1.0 to 1.0, which was represented by a color gradient from light yellow to dark blue. Correlations closer to 1 indicated synchronized residue movements, while correlations closer to −1 indicated opposing movements.

**Figure 12:**
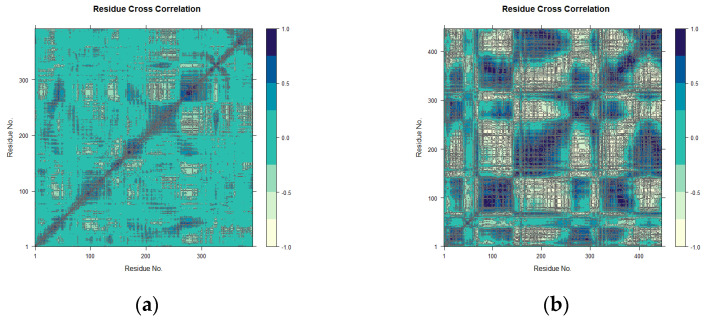
DCCM plots for the complexes. (**a**) mTYR–maculosin; (**b**) TYRP1–maculosin.

DCCM analysis highlighted distinct binding dynamics of maculosin with mTYR and TYRP1. In the maculosin–mTYR complex (Figure 12a), correlated motions were localized to residues 150–200 and 250–300. Molecular docking studies identified key residues such as ASN-260, HIS-263, VAL-283, and ALA-286 in the active site, which exhibited high correlations in the DCCM analysis. This suggests their critical role in the stability and dynamic coupling of the complex. These residues, likely involved in the enzyme’s opening and closing motions, appear to regulate conformational changes in the active site through cooperative interactions, thereby influencing substrate binding and release [44].

In contrast, the maculosin–TYRP1 complex exhibited broader correlated motions across residues 75–150, 200–300, and 350–450 with more extensive correlated and anticorrelated dynamics (Figure 12b). Key residues identified through molecular docking, including GLY-389, TYR-362, SER-394, and HIS-381, also showed high correlations in the DCCM analysis, aligning well with the docking results.

In conclusion, maculosin induced localized interactions in mTYR while enhancing dynamic flexibility and conformational changes in TYRP1. Interactions with residues exhibiting high correlation in both proteins may play a crucial role in the compound’s inhibitory activity, emphasizing protein-specific binding mechanisms that are essential for effective drug design.

#### 2.4.4. Binding Free Energy Evaluation of the Complexes

**Figure 13:**
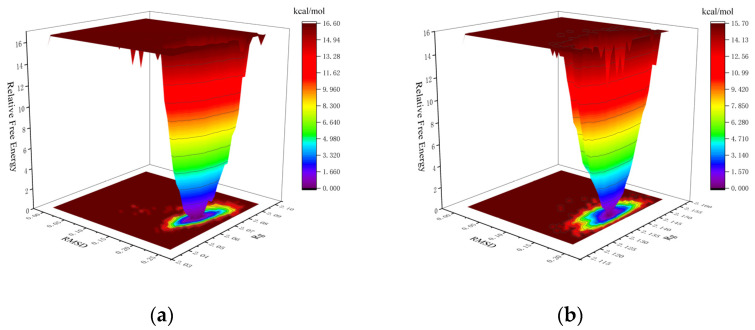
The Gibbs FEL plots of the complex in MD simulations: (**a**) mTYR–maculosin; (**b**) TYRP1–maculosin.

The Gibbs free energy landscape (FEL) was calculated using GROMACS scripts (g_sham and xpm2txt.py) with RMSD and Rg values as input. In the Gibbs FEL plots, RMSD, Rg, and Gibbs free energy are represented on the X, Y, and Z axes, respectively. Weak interactions are depicted as rough, multi-clustered surfaces, whereas strong interactions are characterized by smooth, single-clustered surfaces. Stable conformations are marked in dark purple/blue, while unstable ones appear in red/yellow. In the mTYR–ligand complex, both maculosin (Figure 13a) and tropolone [31] formed a single, dark purple/blue energy cluster, indicating strong and stable interactions with mTYR. Similarly, in the TYRP1–ligand complex, maculosin (Figure 13b) and kojic acid (Figure S3) also formed single, compact energy clusters, suggesting stable interactions between maculosin and TYRP1. The lowest-energy conformations of maculosin in complexes with mTYR and TYRP1 were extracted from the simulation trajectories and are shown in Figures S4 and S5, respectively.

**Figure 14:**
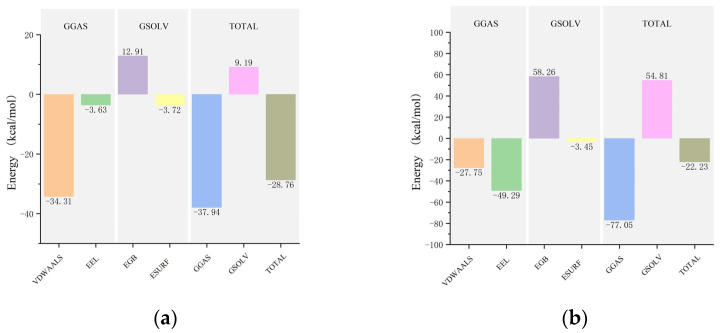
The MM-PBSA binding energy plots of the complex in MD simulations: (**a**) mTYR–maculosin; (**b**) TYRP1−maculosin. In the MD simulations, VDWAALS, EEL, EGB, ESURF, GGAS, GSOLV, and TOTAL represent the following distinct energy components: van der Waals interactions, electrostatic energy, polar and nonpolar solvation energies, molecular mechanics, solvation energy, and average binding free energy, respectively.

After achieving stability in the complex systems, the average binding free energies for the mTYR and TYRP1 complexes with maculosin and co-crystal ligands (tropolone and kojic acid) were calculated using the MM/PBSA method. The average binding free energies for mTYR with maculosin (Figure 14a, Table S3) and tropolone (Table S4) were −28.76 kcal/mol and −12.47 kcal/mol, respectively, indicating a stronger binding affinity of mTYR for maculosin. In the TYRP1–ligand complex, the average binding free energy of maculosin (Figure 14b, Table S5) with TYRP1 was −22.23 kcal/mol compared to −12.73 kcal/mol for kojic acid (Table S6). Overall, maculosin demonstrated stronger binding to the target proteins than the co-crystal inhibitors.

**Figure 15:**
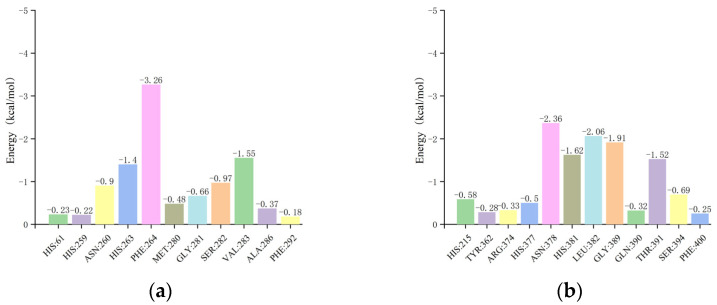
The residue energy plots of the complex in MD simulations: (**a**) mTYR−maculosin; (**b**) TYRP1−maculosin.

In the residue energy analysis of the mTYR–ligand complex, maculosin showed optimal binding with the amino acid residues HIS-263, PHE-264, and VAL-283 in the mTYR protein with binding energies of −1.4, −3.26, and −1.55 kcal/mol, respectively (Figure 15a). The critical roles of VAL-283 and HIS-263 in the interaction between maculosin and mTYR were also corroborated by previous molecular docking simulations. The co-crystal ligand tropolone displayed effective binding to the amino acid residues VAL-283, ASN-260, and HIS-263 with binding energies of −1.59, −1.17, and −1.11 kcal/mol, respectively [41].

For the TYRP1–ligand complex, residue energy analysis revealed that maculosin exhibited optimal binding with ASN-378, HIS-381, LEU-382, GLY-389, and THR-391 in the TYRP1 protein with binding energies of −2.36, −1.62, −2.06, −1.91, and −1.52 kcal/mol, respectively (Figure 15b). Additionally, HIS-381, GLY-389, and THR-391 were identified as critical residues in the TYRP1–maculosin interaction, which was consistent with previous docking studies. Kojic acid showed strong interactions with ASN-378, HIS-381, and THR-391 with binding energies of −1.1, −1.22, and −1.18 kcal/mol, respectively (Figure S6).

In summary, the molecular docking findings for maculosin were further validated through molecular dynamics simulations, demonstrating strong binding stability between maculosin and the target proteins. These results provide compelling evidence supporting maculosin’s observed inhibitory activity against tyrosinase (both mushroom tyrosinase and in B16F10 cells).

## 3. Materials and Methods

### 3.1. Chemicals and Reagents

3-(4,5-Dimethyl-thiazol-2-yl)-2,5-diphenyl tetrazolium bromide (MTT), L-DOPA, and α-melanocyte-stimulating hormone (α-MSH) were purchased from Sigma-Aldrich (St. Louis, MO, USA). Dimethyl sulfoxide (DMSO), phosphate-buffered saline (PBS), and RIPA buffer were obtained from Biosesang (Seongnam-si, Gyeonggi-do, Republic of Korea). Fetal bovine serum (FBS) was supplied by Gibco (Life Technologies, Grand Island, NY, USA). Dulbecco’s Modified Eagle’s Medium (DMEM) and penicillin/streptomycin were purchased from Thermo Fisher Scientific (Waltham, MA, USA). Maculosin was procured from Cayman Chemical Company (Ann Arbor, MI, USA). The protease inhibitor cocktail was obtained from Cell Signaling Technology (Beverly, MA, USA). The bicinchoninic acid (BCA) assay kit was acquired from Thermo Scientific (Waltham, MA, USA) and used according to the manufacturer’s instructions.

### 3.2. Cell Culture and Materials

B16F10 mouse melanoma cells (ATCC) were cultured in DMEM supplemented with 10% fetal bovine serum (FBS) and 1X penicillin/streptomycin (P/S). Cell viability was evaluated using the MTT assay. Cells were seeded at a density of 1.5 × 10^4^ cells/mL in 24-well plates, treated with the samples, and incubated in a 37 °C, 5% CO_2_ atmosphere for 3 d. After 3 h of incubation with 0.5 mg/mL MTT solution, the media was removed. The formed formazan crystals were dissolved in DMSO, and cell viability was quantified by measuring absorbance at 540 nm using a spectrophotometric microplate reader.

### 3.3. Melanin Contents of Maculosin

B16F10 melanoma cells were seeded at a density of 8.0 × 10^4^ cells/well in 60 mm culture dishes. After 24 h, the cells were treated with various concentrations of the sample and α-MSH (100 nM). Following a 72 h incubation, the cells were lysed and centrifuged at 15,000 rpm, −8 °C for 30 min to separate the supernatant. The remaining pellet was used to measure melanin content at 540 nm absorbance.

### 3.4. Tyrosinase Inhibition Activity of Maculosin

The samples at various concentrations were treated with α-MSH (100 nM) and lysed using RIPA buffer containing a protease inhibitor cocktail (1X). The cell lysates were centrifuged, and the supernatant was quantified using the BCA protein assay. After quantification, L-dopa (2 mg/mL in 0.1M sodium phosphate buffer, pH 6.8) was added, and the reaction was incubated in the dark at 37 °C for 1.5 h. Tyrosinase activity was then measured by absorbance at 490 nm.

### 3.5. Statistical Analysis

All experimental data are presented as the mean ± standard deviation (SD) of at least three independent experiments. Statistical analyses were performed using Student’s *t*-test. Statistical significance was determined with *p*-values of * *p* < 0.05, ** *p* < 0.01.

### 3.6. Computational Methodology

#### 3.6.1. Molecular Properties and Drug Likeness

Pharmacokinetic parameters were predicted by assessing the ADMET and drug-likeness properties based on the SMILES data of the compounds. This comprehensive analysis utilized multiple models, including ADMETlab 3.0, SwissADME (http://www.swissadme.ch/; accessed on 20 November 2024), and pkCSM (https://biosig.lab.uq.edu.au/pkcsm/; accessed on 20 November 2024).

#### 3.6.2. Molecular Docking Simulation

The receptor proteins, including mushroom tyrosinase (PDB ID: 2Y9X), human tyrosinase-related protein 1 (PDB ID: 5M8M), and *Bacillus megaterium* tyrosinase (PDB ID: 3NQ1), were retrieved from the Protein Data Bank (PDB), and their 3D structures were downloaded. These protein structures were examined using PyMOL 3.0.3 software in preparation for molecular docking studies. The compounds were drawn in 3D using ACD/ChemSketch Freeware, and their structures were optimized with the MMFF94 force field in OpenBabel software 3.0 to obtain the most stable conformations. AutoDock Tools 1.5.6 was employed to prepare the protein structures by adding hydrogen atoms, and the ligands were processed by hydrogenating and identifying rotatable bonds. Grid parameters for docking were defined based on the co-crystal inhibitor positions within the protein structures. The grid settings were as follows: for mTYR, Center (X, Y, Z) = (−10.2, −30.3, −44.4), Size (X × Y × Z) = (15.0 × 15.0 × 15.0); for TYRP1, Center (X, Y, Z) = (−25.8, −26.1, 22.8), Size (X × Y × Z) = (15.0 × 15.0 × 15.0); and for *Bm*TYR, Center (X, Y, Z) = (−9.2, −14.8, 3.2), Size (X × Y × Z) = (15.0 × 15.0 × 15.0). Semi-flexible docking was carried out using AutoDock Vina 1.2.0 with an exhaustiveness of 25 and the Lamarckian genetic algorithm. Re-docking experiments were performed using the co-crystal ligand from the protein structures, and the root mean square deviation (RMSD) was calculated between the initial and re-docked poses.

#### 3.6.3. Molecular Dynamics (MD) Simulations

The MD simulations of the protein–ligand complexes (mTYR and TYRP1) were performed using GROMACS 2022 and 2021, respectively. The Charmm36 force field was applied for mTYR, Amber 14sb for TYRP1, and GAFF2 for the ligands. The protein–ligand systems were solvated in a periodic boundary box of 1.2 nm using the TIP3P water model, and sodium and chloride ions were added to neutralize the system. The simulation protocol involved three stages: energy minimization using the steepest descent algorithm for 50,000 steps, NVT equilibration at 310 K for 50,000 steps with a timestep of 2 fs, and NPT equilibration at 310 K and 1 atm pressure for another 50,000 steps. After equilibration, a 100 ns production MD simulation was performed without constraints, using a 2 fs timestep and saving coordinates every 10 ps.

#### 3.6.4. PCA and DCCM Analysis

The simulation output was re-centered, and the trajectories were converted into DCD format using VMD. The protein dynamics of the system were analyzed through PCA and DCCM using the Bio3D package in RStudio. The steps involved loading the DCD trajectory data and protein structure, selecting the C_α_ atoms, and aligning the trajectory to the reference protein structure. PCA was performed on the aligned coordinates, and the contributions of the principal components were visualized using color mapping. The DCCM function was used to compute the dynamic cross-correlation matrix based on the C_α_ atom coordinates, and the results were visualized to display dynamic correlations across the protein structure.

## 4. Conclusions

Maculosin, a DKP derivative, has emerged as a promising inhibitor of melanogenesis and tyrosinase activity, as demonstrated through comprehensive in vitro and in silico evaluations. In vitro, maculosin (≤100 µM) did not affect cell viability, but it inhibited melanin production by 14.84% and tyrosinase activity by 19.35%, suggesting its potential to suppress melanogenesis. Compared to other inhibitors, such as tropolone, kojic acid, and arbutin, maculosin demonstrated advantageous ADMET properties, minimal adverse effects on physiological systems, and high drug-likeness potential.

Molecular docking results indicated that maculosin exhibited strong binding affinity with mTYR, TYRP1, and *Bm*TYR. To investigate the inhibitory effects of maculosin on mTYR and melanogenesis and tyrosinase activity in B16F10 cells, we selected mTYR and TYRP1 for MD simulations. The simulation results showed that the complexes formed between maculosin and these two tyrosinases demonstrated good structural stability (RMSD and RMSF), strong binding compactness (Rg), as well as favorable surface hydrophobicity and solvation stability (SASA). Furthermore, the dynamic structures of the complexes maintained good stability throughout the simulation.

The PCA results revealed that the TYRP1 complex displayed greater conformational flexibility and higher mobility compared to mTYR. The DCCM analysis further confirmed that key amino acid residues identified in molecular docking exhibited significant correlated motions during the simulation. Although there were some differences in flexibility between the mTYR and TYRP1 protein complexes, both complexes demonstrated good dynamic stability at the binding sites.

Free energy analysis, including calculations of Gibbs free energy and average binding free energy, revealed that the complexes formed by maculosin and the two protein targets exhibited a single energy cluster in the Gibbs free energy landscape, suggesting that these complexes were thermodynamically stable. The binding stability of the maculosin–protein complexes was comparable to that of known co-crystal inhibitors with superior average binding free energies. Residue energy contribution analysis identified key amino acid residues, including VAL-283 and HIS-263 in mTYR, and HIS-381, GLY-389, and THR-391 in TYRP1, as important contributors to maculosin binding.

In conclusion, the results suggested that maculosin held great potential in inhibiting pigmentation and acting as a skin-whitening agent, making it a promising candidate for further development.

## Data Availability

Data are contained in the article and Supplementary Material.

## Supplementary Materials

The following supporting information can be downloaded at https://www.mdpi.com/article/10.3390/molecules30040860/s1, Table S1. ADMET properties of the compounds; Table S2. Drug-likeness properties of the compounds; Table S3. Binding free energy analysis of the mTYR–maculosin complex using MM/PBSA; Table S4. Binding free energy analysis of the mTYR–tropolone complex using MM/PBSA; Table S5. Binding free energy analysis of the TYRP1–maculosin complex using MM/PBSA; Table S6. Binding free energy analysis of the TYRP1–kojic acid complex using MM/PBSA; Figure S1. Binding interactions of *Bm*TYR protein with kojic acid; Figure S2. The conformational structures of the complex (TYRP1–kojic acid) in MD simulations; Figure S3. The Gibbs FEL plots of the complex (TYRP1–kojic acid) in MD simulations; Figure S4. The lowest energy conformations of the complex (mTYR–maculosin) in MD simulations; Figure S5. The lowest energy conformations of the complex (TYRP1–maculosin) in MD simulations; Figure S6. The residue energy plots of the complex (TYRP1–kojic acid) in MD simulations.
