# Supplementary material for: In Vitro and In Silico Studies of Maculosin as a Melanogenesis and Tyrosinase Inhibitor
Source: Molecules. 2025 Feb 13;30(4):860. doi: 10.3390/molecules30040860 (PMC11858074; doi:10.3390/molecules30040860)

In Vitro and *In Silico* Studies of Maculosin as a Melanogenesis and Tyrosinase Inhibitor in B16F10 Melanoma Cells

Yang Xu [1], Xuhui Liang[1], Hyeon-Mi Kim [1], and Chang-Gu Hyun [1,2*]

[1] Jeju Inside Agency and Cosmetic Science Center, Jeju National University, Jeju 63243, Korea

[2] Department of Beauty and Cosmetology, Jeju National University, Jeju 63243, Korea

* Correspondence: cghyun@jejunu.ac.kr; Tel.: +82-64-754-1803

Supplementary materials

Table S1. ADMET properties of the compounds. 2

Table S2. Drug-likeness properties of the compounds. 3

Table S3. Binding free energy analysis of the mTYR-maculosin complex using MM/PBSA. 3

Table S4. Binding free energy analysis of the mTYR-tropolone complex using MM/PBSA. 3

Table S5. Binding free energy analysis of the TYRP1-maculosin complex using MM/PBSA. 3

Table S6. Binding free energy analysis of the TYRP1-kojic acid complex using MM/PBSA. 3

Figure S1. Binding interactions of BmTYR protein with kojic acid. 4

Figure S2. The conformational structures of the complex (TYRP1-kojic acid) in MD simulations. 4

Figure S3. The Gibbs FEL plots of the complex (TYRP1-kojic acid) in MD simulations. 4

Figure S4. The lowest energy conformations of the complex (mTYR-maculosin) in MD simulations. 5

Figure S5. The lowest energy conformations of the complex (TYRP1-maculosin) in MD simulations. 5

Figure S6. The residue energy plots of the complex (TYRP1-kojic acid) in MD simulations. 5

Table S1. ADMET properties of the compounds.

**ADMET properties**		**Maculosin**	**Kojic acid**	**Tropolone**	**Arbutin**
**Absorption**					
Caco-2 permeability (cm/s) [a]		-0.009	0.637	1.558	0.009
P-gp Ⅰ protein inhibitor [a]		No	No	No	No
P-gp Ⅱ protein inhibitor [a]		No	No	No	No
P-gp substrate [a]		Yes	No	No	No
Human intestinal absorption [a]		66.287%	93.152%	98.108%	38.027%
**Distribution**					
Plasma protein binding[b]		44.6%	23.3%	47.9%	54.2%
Volume distribution (L/kg) [a]		0.204	-0.086	-0.045	0.026
Blood–brain barrier[c]		No	No	Yes	No
**Metabolism**					
CYP450	CYP1A2 inhibitor[c]	No	No	No	No
	CYP2C19 inhibitor[c]	No	No	No	No
	CYP2C9 inhibitor[c]	No	No	No	No
	CYP2D6 inhibitor[c]	No	No	No	No
	CYP3A4 inhibitor[c]	No	No	No	No
**Elimination**					
Clearance rate (mL/min/kg) [a]		0.264	0.638	0.169	0.524
$T_{1/2}$ (h)[b]		1.398	1.827	1.709	2.361
**Toxicity**					
Hepatotoxicity [a]		Yes	No	No	No
Ames toxicity [a]		No	No	No	No
Skin sensitization [a]		No	No	No	No
hERG inhibition [a]		No	No	No	No
LD_{50} of acute toxicity (mol/kg) [a]		1.674	2.037	1.834	1.641

LD_{50}: lethal dose 50; CYP450: cytochrome p450; $T_{1/2}$: time required for the plasma concentration of a drug to decrease by 50%; hERG: human Ether-a-go-go-Related Gene; a: pkCSM; b: ADMETlab 3.0; c: SwissADME.

Table S2. Drug-likeness properties of the compounds.

**Compound**	**MW[a] (g/mol)**	**HBA[a]**	**HBD[a]**	**RB[a]**	**TPSA[a] (Å^2)**	**Log *P*[a]**	**MR[b]**	**RO5[b]**	**Ghose Filter[b]**	**Veber rule[b]**	**Egan rule[b]**	**Drug likeness**
Maculosin	260	5	2	2	69.64	0.316	76.78	Yes	Yes	Yes	Yes	Yes
Kojic acid	142	4	2	1	70.67	-0.56	33.13	Yes	No*	Yes	Yes	No
Tropolone	122	2	1	0	37.3	0.667	34.74	Yes	No*	Yes	Yes	No
Arbutin	272	7	5	3	119.61	-0.996	62.61	Yes	No**	Yes	Yes	No

MW: molecular weight; HBA: number of H-Bond acceptors; HBD: number of H-Bond donors; RB: number of rotatable bonds; TPSA: Topological Polar Surface Area; MR: molar refractivity; a: ADMETlab 3.0; b: SwissADME; *: three violations: MW < 160, MR < 40, and atoms < 20; **: one violation: WLOGP<-0.4.

Table S3. Binding free energy analysis of the mTYR-maculosin complex using MM/PBSA.

Frames	VDWAALS	EEL	EGB	ESURF	GGAS	GSOLV	TOTAL
Average	–34.31	–3.63	12.91	–3.72	–37.94	9.19	–28.76
SD	5.23	2.41	2.65	0.47	6.65	2.31	4.80
SEM	0.94	0.43	0.48	0.08	1.19	0.41	0.86

Table S4. Binding free energy analysis of the mTYR-tropolone complex using MM/PBSA.

Frames	VDWAALS	EEL	EGB	ESURF	GGAS	GSOLV	TOTAL
Average	–18.24	–5.63	14.24	–2.84	–23.86	11.40	–12.47
SD	1.46	2.53	1.78	0.10	2.66	1.79	1.50
SEM	0.19	0.32	0.23	0.01	0.34	0.23	0.19

Table S5. Binding free energy analysis of the TYRP1-maculosin complex using MM/PBSA.

Frames	VDWAALS	EEL	EGB	ESURF	GGAS	GSOLV	TOTAL
Average	–27.45	–49.29	58.26	–3.45	–77.05	54.81	–22.23
SD	2.00	8.80	7.36	0.18	8.02	7.30	1.91
SEM	0.36	1.58	1.32	0.03	1.44	1.31	0.34

Table S6. Binding free energy analysis of the TYRP1-kojic acid complex using MM/PBSA.

Frames	VDWAALS	EEL	EGB	ESURF	GGAS	GSOLV	TOTAL
Average	–18.17	–2.74	11.14	–2.96	–20.91	8.18	–12.73
SD	2.48	6.68	4.15	0.28	5.79	4.24	3.65
SEM	0.45	1.20	0.74	0.05	1.04	0.76	0.66

Figure S1. Binding interactions of BmTYR protein with kojic acid.

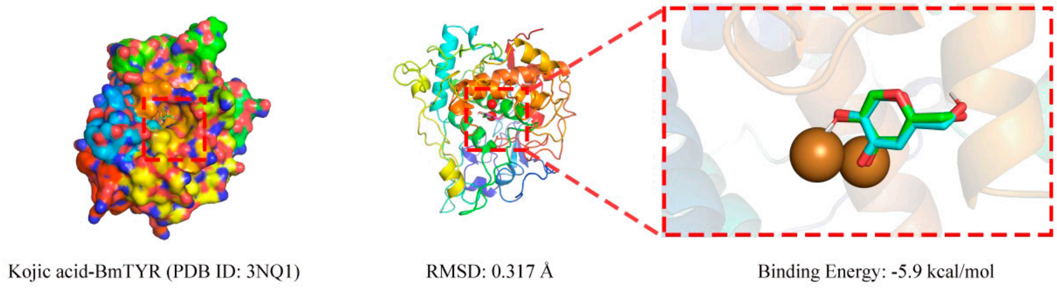

Figure S2. The conformational structures of the complex (TYRP1-kojic acid) in MD simulations.

The configurations at different time points are represented as follows: 0 ns (green), 25 ns (blue), 50 ns (purple), 75 ns (yellow), and 100 ns (pink).

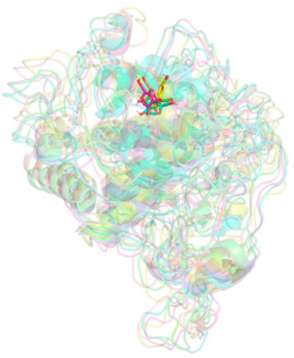

Figure S3. The Gibbs FEL plots of the complex (TYRP1-kojic acid) in MD simulations.

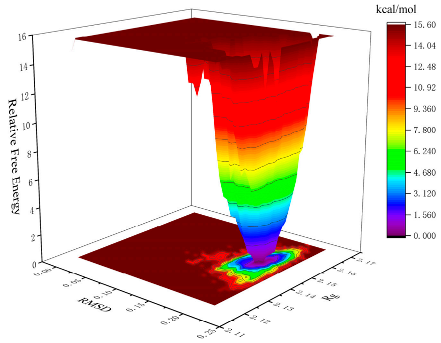

Figure S4. The lowest energy conformations of the complex (mTYR-maculosin) in MD simulations.

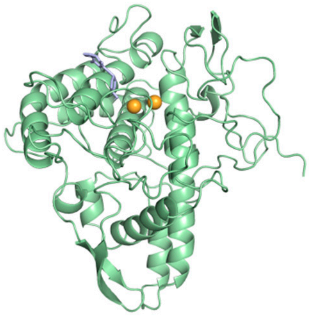

Figure S5. The lowest energy conformations of the complex (TYRP1-maculosin) in MD simulations.

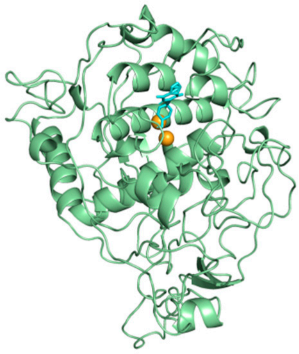

Figure S6. The residue energy plots of the complex (TYRP1-kojic acid) in MD simulations.

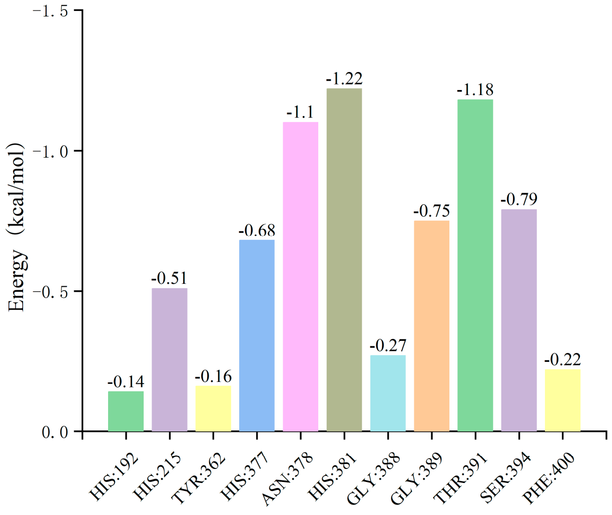

Supplement: Supplementary file 1 [file molecules-30-00860-s001.zip › molecules-3380651-supplementary.pdf]
